# Full genome characterization of porcine circovirus type 3 isolates reveals the existence of two distinct groups of virus strains

**DOI:** 10.1186/s12985-018-0929-3

**Published:** 2018-01-29

**Authors:** Robert Fux, Christina Söckler, Ellen Kathrin Link, Christine Renken, Roman Krejci, Gerd Sutter, Mathias Ritzmann, Matthias Eddicks

**Affiliations:** 1 Institute for Infectious Diseases and Zoonoses, LMU Munich, Veterinärstrasse 13, 80539 Munich, Germany; 2 Clinic for Swine at the Centre for Clinical Veterinary Medicine, LMU Munich, Sonnenstrasse 16, 85764 Oberschleissheim, Germany; 3 CEVA, La Ballastiere - BP 126, 33501 Libourne, France

**Keywords:** PCV3, Porcine circovirus, Swine pathogen, Emerging disease

## Abstract

**Background:**

The occurrence of the novel porcine circovirus type 3 (PCV3) was reported from the Americas, Asia and Europe. Although this virus was detected in association with various clinical syndromes in pigs, its role as possible swine pathogen remains unclear. PCV3 was detected with high prevalence in Polish farms, but to date no genome sequences were available from European PCV3 strains.

**Methods:**

We collected 1060 serum samples from piglets at the age of 20–24 weeks from 53 farms distributed all over Germany. PCV3 DNA was detected using a real-time PCR and subsequently complete PCV3 genome sequences were obtained after multiply primed rolling circle amplification and sequencing of overlapping PCR products. Phylogenetic analysis was performed by neighbor-joining method and maximum likelihood method.

**Results:**

We obtained 15 complete PCV3 genome sequences as well as nine partial sequences including the putative ORFs 1, 2 and 3 from PCV3 viremic animals in German pig farms. Phylogenetic analysis of these German as well as 30 full genome sequences received from GenBank divided the PCV3 strains into two main groups and several subclusters. Furthermore, we were able to define group specific amino acid patterns in open reading frame 1 and 2.

**Conclusion:**

PCV3 is distributed with high prevalence in German pig industry. Phylogenetic analysis revealed two clearly separated groups of PCV3 strains, which might be considered as PCV3 genotypes. Specific nucleotide and amino acid marker positions may serve for easy and fast intraspecies classification and genotyping of PCV3 strains. No correlation between PCV3 variants with their geographical origin was evident. We found the same diversity of PCV3 strains in Germany as in other countries. We hypothesize that PCV3 is not a newly emerging virus in the German pig population. Future studies will have to show, if PCV3 genotype specific biological properties are evident.

**Electronic supplementary material:**

The online version of this article (10.1186/s12985-018-0929-3) contains supplementary material, which is available to authorized users.

## Background

The discovery of a new virus species in humans as well as in animals always raises the same questions. What is its pathogenic potential? Are specific diseases or symptoms triggered by the virus? What is the prevalence in the susceptible population? Is it a newly emerging virus? Is it a homogeneous virus population or are there different virus variants? Recently, a new type of porcine circoviruses (PCV) was described, and consequently the name porcine circovirus type 3 (PCV3) was proposed. First discovered in the USA [1, 2], there are now several reports or sequences available from China [3], South Korea [4], Poland [5] and Brazil [6]. The detection of PCV3 was associated with different clinical syndromes and diseases in pigs of different ages. The porcine dermatitis and nephropathy syndrome [1] was noticed as well as reproductive problems [1, 3, 6], cardiac and multisystemic inflammation [1], respiratory diseases [7] and congenital tremors in neonatal pigs [8]. However, high PCV3 prevalence was also reported from randomly selected farms of different health status from Poland without association of PCV3 to specific clinical signs [5]. Similar results were obtained by a study conducted in Korea [4].

**Fig. 1:**
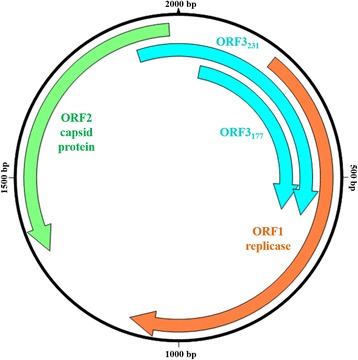
Schematic presentation of the PCV3 genome and its putative open reading frames (ORF)

PCV1 was first identified as cell culture contaminant in the 1980s and is considered as nonpathogenic for pigs [9]. In contrast, PCV2 is associated with several clinical diseases and syndromes and is responsible for major economic losses in swine industry worldwide [10]. All three PCVs are small, non-enveloped viruses with a circular, single-stranded DNA genome. The genome size of PCV1 is about 1760 bp, the typical PCV2 genome varies, depending on the genotype, between 1767 bp (PCV2b, 2c and 2d) and 1777 bp (PCV2e) and PCV3 has the largest genome with 2000 bp (see Fig. 1). Two major open reading frames (ORF) encode the replicase protein (ORF1) and the capsid protein (ORF2), respectively. In contrast to PCV1 and PCV2 a canonical start codon (ATG) is missing in ORF1 of PCV3. Here an alternative start codon was discussed, as it has been proposed for some avian circoviruses [2]. The PCV3 ORF2 is in opposite orientation to ORF1 and encodes a 214 amino acid (aa) protein. For PCV2 two further ORFs were characterized as apoptosis-inducing (ORF3) and apoptosis-suppressing gene (ORF4) (for review see [11]). Also for PCV3 a third putative ORF was described. Similar to PCV3 ORF1 the start codon for ORF3 remains unclear. An alternative initiation codon (TCG, nucleotide (nn) position 1900–1902) would result in a 231 aa protein (ORF3_231_), whereas a methionine codon (ATG, nn position 62–64) would yield a 177 aa protein (ORF3_177_) [2].

Based on phylogenetic analysis and the definition of genotype-specific marker positions four PCV2 genotypes (2a-2d) could be defined by Franzo and colleagues [12]. Furthermore, a fifth genotype (2e) was reported from the USA [13]. The correct taxonomical classification and genotyping is important for molecular epidemiology. However, the precise classification of virus strains within the species PCV2 has been the occasion for several controversial discussions (for review see [12]). Because of the very limited number of available PCV3 sequences or the use of small fragments of the PCV3 genome, results of performed phylogenetic analysis of PCV3 has to be considered as preliminary [3, 4].

In this study we report about (i) the first record of PCV3 in Germany, (ii) the PCV3 prevalence in German swine farms, (iii) the phylogenetic analysis of German PCV3 sequences, which suggested a clear division of PCV3 strains into two groups and (iv) the identification of group specific marker codons.

## Methods

### Sample origin

The examined sera were obtained within a serological porcine health screening (approved by the ethic committee of the Centre for Clinical Veterinary Medicine, approval number 73–31–05-2016) in 53 German fattening farms with noticeable respiratory clinical signs from 2015 (Table 1). The age of all sampled pigs ranged between 20 and 24 weeks. On each farm 20 serum samples were collected. In detail, 23 farms were located in Southern Germany (Bavaria or Baden-Wuerttemberg), 10 farms in Eastern Germany (Mecklenburg-West Pomerania, Brandenburg, Saxony-Anhalt or Thuringia) and 20 farms in Northern Germany (Lower Saxony, North Rhine-Westphalia or Schleswig-Holstein). Herd sizes varied between 400 and 28.000 animals. Piglet producing farms were located in Germany, Denmark and The Netherlands.

**Table 1:** Details of 53 sampled farms

Farm	Location	Origin	Herd size
1	E	D	3
2	E	E	3
3	E	E	2
4	N	D	2
5	N	N	2
6	E	E	3
7	E	NL	2
8	S	E	2
10	E	D	4
12	E	E	4
13	N	E	2
14	N	N	2
15	N	E	2
16	N	N	3
17	N	NL	4
18	S	S	1
19	S	S	1
20	S	S	2
21	S	S	1
22	S	S	2
23	S	S	2
24	N	N	2
25	N	N	2
26	S	S	2
27	S	S	1
28	S	S	2
29	S	S	1
30	S	E	3
31	S	S	1
32	S	E	1
33	S	S	2
34	S	S	2
35	S	D	3
36	E	E	2
37	S	S	2
38	S	S	2
39	N	N	2
40	N	N	2
41	N	N	2
42	N	N	2
43	N	N	2
44	N	N	1
45	N	NL	2
46	N	N	2
47	S	N	2
48	S	N	1
49	N	D	2
50	N	E	2
51	N	N	2
52	E	N	2
53	S	S	2
54	S	S	1
55	E	D	3

*Location* location of farm, *Origin* location of piglet producing farm, *S* Southern Germany, *E* Eastern Germany, *N* Northern Germany, *D* Denmark, *NL* The Netherlands, Herd size: 1 = < 1000 pigs, 2 = 1000–4000 pigs, 3 = 4001–10,000 pigs, 4 = > 10,000 pigs. (Farms 9 and 11 were not included in this study)

### PCV3 detection

PCV3 DNA was detected using a real-time PCR assay published previously by Palinski and colleagues [2]. First, serum pools (*n* = 5) were screened for PCV3. If a sample pool was PCV3 DNA positive, the included single samples were tested subsequently. If two or more serum pools from one farm were tested positive, only the pool with the lowest Cq (quantification cycle) value (indicating highest viral load) was investigated further. In total, we tested 212 serum pools and 200 single samples.

DNA from serum samples was isolated using the NucleoSpin Virus Core Kit (Macherey-Nagel) and a Microlab Starlet workstation (Hamilton Robotics) according to the manufacturer’s instruction. For the real-time PCR we used the QuantiTect probe PCR kit (Qiagen). Oligonucleotide primers (PCV3-F: 5′ AGT GCT CCC CAT TGA ACG; PCV3-R: ACA CAG CCG TTA CTT CAC) were used with a final concentration of 800 nM, the TaqMan probe (5’ FAM-ACC CCA TGG CTC AAC ACA TAT GAC C-BHQ1) was used with 200 nM. The thermal profile of the PCR was: 94 °C for 15 min, and 42 cycles of 94 °C for 15 s and 60 °C for 60 s.

### Sequencing of PCV3 DNA

Because of the high Cq values of many samples, indicating a low viral load, we performed a multiply primed rolling circle amplification (RCA) prior to amplification of PCR products for sequencing. For RCA we used the TempliPhi 100 amplification kit (GE Healthcare) according to the manufacturer’s protocol. Briefly, 1 μl of isolated DNA was mixed together with 5 μl of TempliPhi sample buffer. Then, the preparation was denatured at 95 °C for 3 min and cooled down to room temperature. 5 μl of TempliPhi reaction buffer and 0.2 μl of TempliPhi enzyme mix was added and the reaction was run overnight at 30 °C. The next day, the φ29 DNA polymerase was inactivated at 65 °C for 10 min and samples were stored at − 20 °C until further usage.

To obtain the complete PCV3 genome sequence, we amplified three overlapping PCR products (PCV3_74–1144_, PCV3_1137–1561_ and PCV3_1427–433_). Therefore, we modified the primer setup initially described by Paliski and colleagues [2] (see Table 2). We used 1 μl of the RCA reaction as template for the PCRs performed with the Q5 High Fidelity PCR Kit (New England Biolabs) using the following thermal profile: 98 °C for 5 min, and 30 cycles of 98 °C for 30 s, 55 °C for 60 s and 72 °C for 2 min. PCR products were controlled by agarose gel electrophoresis and sequenced using the PCR primers (Table 2) and the sequencing service of Eurofins Genomics (Ebersberg, Germany).

**Table 2:** Oligonucleotide primers used for amplification and sequencing PCV3 genome fragments

Primer	Sequence 5′-3′	PCR product
PCV3_74_ F	CAC CGT GTG AGT GGA TAT AC	1072 bp
PCV3_1144_ R	CAC CCC AAC GCA ATA ATT GTA	
PCV3_910_ F^a^	GAC AAT TCC CAC CCA AAC	
PCV3_1137_ F	TTG GGG TGG GGG TAT TTA TT	425 bp
PCV3_1561_ R	ACA CAG CCG TTA CTT CAC	
PCV3_1427_ F	AGT GCT CCC CAT TGA ACG	1007 bp
PCV3_433_ R	CGA CCA AAT CCG GGT AAG C	

^a^only used for sequencing

### Sequence assembly and analysis

DNASTAR Lasergene and MEGA6 software was used for assembly, alignment and analysis of the sequences. A clustalW algorithm was used for alignments, and phylogenetic trees (based on the full genome sequence or the ORF2) were constructed using the neighbor-joining (NJ) method (p-distance model, 1000 bootstraps) and the maximum likelihood method. For comparison with the German PCV3 sequences, all currently available complete PCV3 genomes (from Brazil, China, South Korea and USA) were downloaded from NCBI GenBank (see Additional file 1).

### Statistic examination

For the statistical analysis we used SPSS 23 for windows (IBM® SPSS Inc., USA). Each single farm or each sequenced strain served as a statistical unit, respectively. The significance level of this investigation was 5% with a confidence interval of 95%. To evaluate a possible association between the farm specific factors “*origin of fattening pigs*”, “*location of fattening farm*” and “*herd size of fattening farm*” (Table 1) and the occurrence of PCV3 or an assumed PCV3-cluster on a farm, we used cross tables and Yates’ chi-squared test.

## Results

### PCV3 Prevalence in German pig farms

We investigated 53 German farms of different location and size for the occurrence of PCV3 viremic fattening pigs. In 75% (40/53) of the farms we detected PCV3 DNA at least in one serum pool (Table 3). Cq values of pool samples ranged from 26 to 39. Neither the location of the farm, the origin of the piglets nor the herd size had a significant influence on virus occurrence on farm level.

**Table 3:** PCV3 real-time PCR results and PCV3 sequences

Farm	PCV3 positive pools	Range of Cq values	Obtained PCV3 sequence
1	0	NA	NA
2	3/4	33–36	DE2.8p
3	2/4	30–33	DE3.7c
4	2/4	31–33	DE4.3c
5	1/4	32	DE5.15p
6	3/4	32–36	DE6.1p
7	3/4	26–38	DE7.3c
8	1/4	32	NA
10	2/4	34–37	NA
12	3/4	27–35	DE12.19p
13	3/4	35–38	DE13.20c
14	3/4	33–36	DE14.15p
15	2/4	32–38	DE15.19p
16	0	NA	NA
17	1/4	37	DE17.20p
18	2/4	31	DE18.2c
19	1/4	32	DE19.15c
20	4/4	33–38	NA
21	0	NA	NA
22	1/4	31	NA
23	2/4	31–33	DE23.17c
24	2/4	37–38	NA
25	0	NA	NA
26	3/4	27–35	DE26.17c
27	1/4	31	DE27.16c
28	3/4	33–37	DE28.12p
29	1/4	36	NA
30	1/4	38	NA
31	2/4	32–37	DE31.17p
32	0	NA	NA
33	0	NA	NA
34	2/4	32–35	DE34.5c
35	1/4	36	NA
36	0	NA	NA
37	1/4	38	NA
38	0	NA	NA
39	1/4	37	NA
40	1/4	39	NA
41	1/4	30	DE41.16c
42	0	NA	NA
43	0	NA	NA
44	3/4	32–36	NA
45	0	NA	NA
46	3/4	35–36	NA
47	0	NA	NA
48	2/4	31–37	DE48.7c
49	2/4	33–38	NA
50	0	NA	NA
51	1/4	34	NA
52	2/4	34–36	DE52.18c
53	3/4	28–34	DE53.8c
54	2/4	35–36	NA
55	2/4	34–37	DE55.1c

*c* complete PCV3 genome sequence, *p* partial PCV3 genome sequence (ORFs 1, 2 & 3 complete), *NA* not adequate, (Farms 9 and 11 were not included in this study)

### Characterization of German PCV3 genome sequences

We used selected single serum samples (with the lowest Cq values) for amplification and sequencing of the PCV3 genome. We obtained 15 full genome sequences and nine partial sequences, including all three putative ORFs, representing 24 of the 40 PCV3 positive farms (see Table 3). As described by Palinsky and colleagues (2017), in 13 cases the amplified and sequenced PCV3 genome fragments assembled to a 2000 bp long circular genome. However, because of a deletion at nn position 1224 in the noncoding region between ORF1 and ORF2, the genome of strain DE41.16 had a length of only 1999 bp. In contrast to that strain DE26.17 showed an insertion between nucleotide 6 and 7 and a genome length of 2001 bp. This insertion would result in a codon shift of the putative ORF3_231_ starting at nn position 1900 and a stop codon at aa position 47. Additionally, the ORF3_231_ sequence of strain DE15.17 had a stop codon at aa position 21. Therefore, these both virus variants would not be able to induce the synthesis of a functional ORF3_231_ protein. The alternative ORF3_177_, starting at nn position 62, would not be affected by these mutations.

### Phylogenetic analysis of German PCV3 genome sequences

**Fig. 2:**
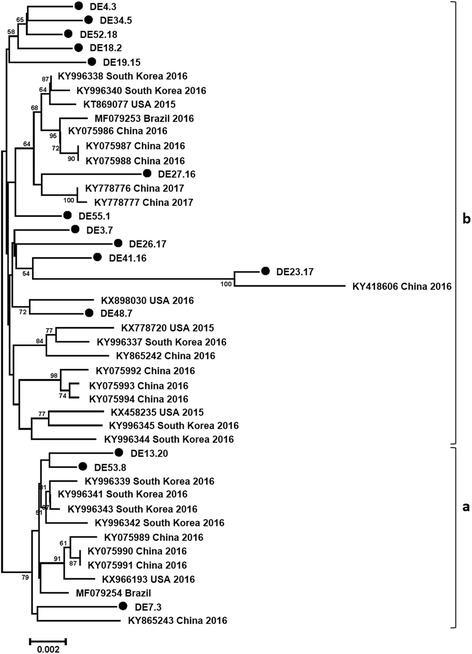
Phylogenetic analysis of PCV3 based on the complete genome of 15 German PCV3 strains (●) and 30 PCV3 reference strains (GenBank accession number, country and year of collection; more details are listed in Additional file 1). The tree was constructed using the neighbor joining method (p-distance model; 1000 bootstraps; only bootstrap values above 50 are shown). The scale bar indicates nucleotide substitutions per site

A NJ tree based on the PCV3 full genome including 15 German and 30 reference sequences is shown in Fig. 2. The sequences could be divided into two main groups (*a* and *b*). A phylogenetic tree constructed with the maximum likelihood (ML) method displayed the same topology (data not shown). In contrast to group *a*, which showed high sequence identities (99.1–100%), group *b* was further subdivided into three clusters and sequence identities were lower (97.3–100%) in this branch.

**Fig. 3:**
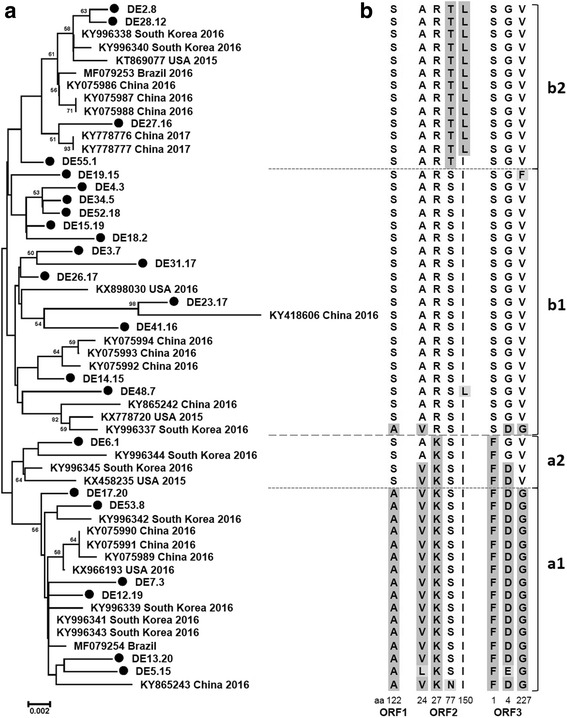
Phylogenetic analysis of PCV3 based on ORF2 and identification of group specific amino acid motifs. **a** Phylogenetic tree based on ORF2 of 24 German PCV3 strains (●) and 30 PCV3 reference strains (GenBank accession number, country and year of collection; more details are listed in Table A1). The tree was constructed using the neighbor joining method (p-distance model; 1000 bootstraps; only bootstrap values above 50 are shown). The scale bar indicates nucleotide substitutions per site. **b** Amino acid alignments of the putative ORFs 1, 2 and 3 were used to identify **group specific motifs**

A corresponding phylogenetic tree including 24 German and 30 reference sequences was constructed based on the nucleotide sequence of the ORF2 (Fig. 3a). Again the two main groups were present. However, three sequences (KX458235, KY996344 and KY96345), which were assigned to group *b* in the full genome based tree, were now allocated together with the German sequence DE6.1 as a separate subcluster in group *a* (*a*2). Interestingly, this subcluster was split when the aa sequence of ORF2 was used for analysis (NJ and ML, data not shown). Strains KX458235 and KY996345 were located within subgroup a1, KY996344 was clustered at the edge of that branch and strain DE6.1 became member of group *b*.

To support the definition of these two main groups and further subgroups, we aligned the putative PCV3 ORFs and identified group specific marker codons (Fig. 3b and Table 4). Because of the overlap of the putative ORF3_231_ with ORF1 and ORF2 (see Fig. 1) three nucleotide changes (nn position 579, 1901 and 1910) resulted in aa alterations in ORF1/2 and ORF3 simultaneously.

**Table 4:** Codon variants which allow PCV3 subtyping (in brackets the proportion of sequences with fitting marker position)

Codon	PCV3 group *a*1	PCV3 group *b*1	PCV3 group *b*2
ORF1–122	**G**CG, alanine (16/16)	**T**CG, serine (20/21)	**T**CG, serine (13/13)
ORF2–24	G**T**C, valine (15/16)	G**C**C, alanine (20/21)	G**C**C, alanine (13/13)
ORF2–27	A**A**A, lysine (16/16)	A**G**A/**CG**A, arginine (21/21)	A**G**A/**CG**A, arginine (13/13)
ORF2–77	A**G**C, serine (15/16)	A**G**C, serine (21/21)	A**C**C, threonine (13/13)
ORF2–150	**A**TT, isoleucine (16/16)	**A**TT, isoleucine (20/21)	**C**TT/**C**T**A**, leucine (12/13)
ORF3_231_–1	T**T**T, phenylalanine (16/16)	T**C**T/T**CG**, serine (21/21)	T**C**T/T**CG**, serine (13/13)
ORF3_231_–4	G**A**C, aspartic acid (15/16)	G**G**C, glycine (20/21)	G**G**C, glycine (13/13)
ORF3_231_–227	G**G**C, glycine (16/16)	G**T**C, valine (19/21)	G**T**C, valine (13/13)

Summarizing the marker codons in ORF1 and ORF2 resulted in a specific aa pattern for group *a* (A V K S I; motif 1) and for group *b* (S A R S I; motif 2) (see Fig. 3b). However, the members of the above-mentioned subgroup (*a*2 in Fig. 3, clustering differently in the ORF2 and the full genome based phylogenetic analysis) demonstrated aa motifs (S V/A K S I) that were between motif 1 and motif 2. Additionally, a clearly separated cluster in group *b* (*b*2) showed a slight modification in motif 2 (S A R **T L**). Therefore, we could define two main groups *a* and *b*, which could be subdivided into two subgroups (*a*1 and *a*2; *b*1 and *b*2), respectively. While nn sequence identities of ORF2 in subgroups *a*1, *a*2 and *b*2 were high (98.0–100%), subgroup *b*1 showed a higher divergence (sequence identity 96.4–99.8%). None of the clusters was significantly correlated to a specific origin of the included samples. PCV3 sequences from Asia, America and Germany were members of all subgroups, respectively.

## Discussion

Recently, several studies with varying approaches demonstrated the existence of PCV3 in Asia, Europe and the USA. Specific specimens from diseased pigs from single farms were investigated as well as random samples from regional screening programs. Organs and tissue samples were used as well as serum samples and oral-fluids. As basis for the design of this study, we hypothesized PCV3 might show similar infection dynamics on farm level like PCV2. Therefore, we used serum samples of piglets after the decline of maternal antibodies, expecting higher rates of viremic animals. This is consistent with studies performed by Kwon and colleagues [4] and Stadejek and colleagues [5], which demonstrated high PCV3 prevalence in weaned pigs. In order to obtain an authentic picture of the PCV3 situation in Germany, the origin of samples represented the main areas with swine production in Germany. Also different structures of pig production in Germany (e.g. herd sizes) were considered. Actually, the sampling was performed to evaluate the seroprevalence of respiratory pathogens in German pig industry. However, our study is not suitable to correlate the presence of PCV3 with the appearance of respiratory diseases in swine. To our opinion, for this purpose the same requirements would have to be fulfilled as for the definition of PCV associated diseases in the context of PCV2: the explicit description of the clinical disease, combined with a (specific) pathohistological picture and the detection (and if possible quantification) of the virus in association with the lesions. To our opinion, the presence of PCV3 and simultaneous absence of other detectable pathogens in a clinical case might indicate an etiological role of PCV3, but more detailed investigations will be essential to identify its real pathogenic potential.

In our study 79 sample pools from 40 farms were positive for PCV3 DNA. Therefore the virus prevalence was 75% at herd level. Similar high values were reported by others. In a study from Poland PCV3 was detected in 12 of 14 farms (86%) [5], in South Korea 53 of 73 farms (73%) were tested PCV3 positive [4] and from China a prevalence of 69% (24 of 35 farms) was reported [3]. The herd size, the geographical location of the farm or the piglet producing unit had no effect on the PCV3 prevalence. Furthermore, no significant correlation between PCV3 detection and PCV2 prevalence and PCV2 vaccination status of the herds was observed, respectively. Therefore, differences in PCV3 prevalence between farms may be attributed to individual factors like pig flow management, biosecurity, immunosuppressing effects or existing herd immunity as it was discussed already by others [5].

Although we performed no quantification of PCV3, the high Cq values of most samples suggest that viral loads in the blood of the viremic animals was moderate or low. Only in 18 (of 200) single tested samples we obtained Cq values < 30. This observation is in consistence with the study from Poland [5]. For PCV2 it is generally accepted, that there is a strong correlation between viral loads in serum or tissues and the severity of virus-induced histopathological lesions (for review see [10]). In regard of the high PCV3 prevalence, the observed low virus loads in this and other studies and the unclear role of PCV3 as possible swine pathogen, the same might be true for PCV3 infections.

Because of the low viral loads of many samples and the moderate sensitivity of the used PCR, we performed RCA prior to amplification of larger fragments of the PCV3 genome for sequencing. Thus, we were able to obtain 15 complete PCV3 genome sequences. Additionally, from other nine strains we were able to determine the sequences of ORF1, ORF2 and ORF3_231_, respectively. Most sequences matched previous descriptions [1, 2]: a circular 2000 bp genome with ORFs for a replicase protein (nn 216–1107) and the capsid protein (nn 1336–1980) and two putative variants of ORF3 (ORF3_231_ nn 1900–595; ORF3_177_ nn 62–595). However, one sequence (DE41.16) had a deletion in the noncoding region between ORF1 and ORF2, resulting a genome length of 1999 bp and another sequence (DE26.17) had a nucleotide insertion between nn position 6 and 7 resulting in a 2001 bp genome sequence. Interestingly, the second mutation would cause a codon shift in the putative ORF3_231_ with a termination signal after aa 47. Additionally, the sequence DE15.17 possessed a stop codon in the amino-terminal part of ORF3_231_. Therefore, both virus variants would be unable to synthesize a 231 aa ORF3 protein. However, the translation of the shorter ORF3_177_ would not be affected. This finding questions the meaning of the large ORF3 and might favor the existence of an ORF3_177_.

The intraspecific classification and genotyping of PCV2 is an ongoing challenge. At the beginning the limited number of published genome sequences was a major problem and nowadays the occurrence of a significant number of recombinant sequences and different rates of evolution within the different clades of the PCV2 phylogenetic tree poses problems to the used PASC (pairwise sequence comparisons) analysis [12] (Franzo et al., 2015). Keeping this and the low number of available PCV3 genome sequences in mind, the authors are aware that the performed phylogenetic analysis will have some kind of preliminary character. However, using the full genome as well as the ORF2 sequences and calculating phylogenetic trees with the neighbor-joining method as well as with the maximum likelihood method, the PCV3 strains were clearly separated into two major clades, which might be considered as two different genotypes of PCV3. Similar was reported by Ku and colleagues [3], however, one reference sequence (MO2015 from USA, KX778720) clustered differently in comparison to our analysis. This might be explained by the small number (ten) of included PCV3 full genome sequences in that work. By analogy to the PCV2 field, we identified marker nucleotide and codon positions, which substantiate the definition of the two PCV3 subgroups. In ORF1 codon 122 and in ORF2 codons 24, 27, 77 and 150 gave a typical aa motif for group *a* (A V K S I) and for group *b* (S A R S I), which might be helpful for the intraspecific classification. Interestingly, a small branch of three or four sequences (*a*2 in Fig. 3) clustered differently in the ORF2 nn, ORF2 aa and the full genome based phylogenetic tree. In addition, the signal motif of these PCV3 viruses represented an intermediate sequence (S A/V K S I) of pattern 1 and 2. These strains, as well as sequence KY966337 (A V R S I), might represent some kind of evolutionary linker between the two main groups or recombinant viruses, although this was not evident. The same might apply to the sequences DE48.7, DE55.1 which show slightly modified signal motifs and may represent some kind of linker between subgroups *b*1 and *b*2. Sequence identities in group *b* were lower than in group *a* and sequences of a separated branch (*b*2 in Fig. 3) demonstrated a typical variance of the motif 2: S A R **T L**. Therefore, the main groups (especially group *b*) might be divided in several subgroups, however, more PCV3 sequences are needed to endorse this assumption. Future work has to demonstrate if these genetic differences correlate to specific biological properties of the PCV3 groups. However, this might be a challenging task, because attempts to isolate infectious PCV3 were unsuccessful up till now, and as for PCV2 it might be difficult to establish appropriate animal models.

One subject of our study was to identify a putative correlation of PCV3 variants with their geographical origin. However, neither for Germany nor for the internationally available sequences this was successful. The German PCV3 sequences were evenly distributed over the main groups and subclusters of the phylogenetic trees and every group or subclade contained sequences from America, Europe and Asia. Although the number of included sequences was limited, these findings indicate a uniform distribution of different PCV3 strains worldwide. The example of the emerging PCV2 genotype 2d shows how fast the worldwide distribution of a new type of PCV can happen [14, 15]. Nevertheless, the available phylogenetic and epidemiological data allow the speculation, that PCV3 is not a newly emerging pathogen, but might be present in the world’s swine population for a longer period.

## Conclusion

We demonstrated that PCV3 is distributed with high prevalence in German pig industry. Phylogenetic analysis of German and international genome sequences revealed two clearly separated groups of PCV3 strains, which might be considered as PCV3 genotypes. Specific nucleotide and amino acid marker positions in ORF 1 and 2 should be useful for intraspecies classification and genotyping of PCV3 strains. To identify biological properties of putative PCV3 genotypes will be a task for future research. A correlation between PCV3 variants with their geographical origin was absent. In Germany the same diversity of PCV3 strains was noticed as in other countries. We suggest that PCV3 is not a newly emerging virus in the German pig population.

## Additional file

Additional file 1: PCV3 sequences used for phylogenetic analysis. (DOCX 15 kb)

## Abbreviations

aa: Amino acid(s)
bp: Base pair(s)
Cq: Quantification cycle
NJ: Neighbor-joining
nM: Nanomolar
nn: Nucleotide(s)
ORF: Open reading frame
PASC: Pairwise sequence comparisons
PCV: Porcine circoviruses
PCV3: Porcine circovirus type 3
RCA: Rolling circle amplification
