# Supplementary material for: Full genome characterization of porcine circovirus type 3 isolates reveals the existence of two distinct groups of virus strains
Source: Virol J. 2018 Jan 29;15:25. doi: 10.1186/s12985-018-0929-3 (PMC5789634; doi:10.1186/s12985-018-0929-3)
Supplement: Additional file 1: — PCV3 sequences used for phylogenetic analysis. (DOCX 15 kb) [file 12985_2018_929_MOESM1_ESM.docx]

**Additional file 1**

**PCV3 sequences used for phylogenetic analysis.**

| **GenBank** | **Country** | **Collection Date** | **ID** |
| --- | --- | --- | --- |
| MF079253 | Brazil | 2016 | BR/RS/6 |
| MF079254 | Brazil | 2016 | BR/RS/8 |
| KY075986 | China | 2016 | CN/Fujian-5/2016 |
| KY075987 | China | 2016 | CN/Fujian-12/2016 |
| KY075988 | China | 2016 | CN/Henan-13/2016 |
| KY075989 | China | 2016 | CN/Jiangxi-62/2016 |
| KY075990 | China | 2016 | CN/Chongqing-147/2016 |
| KY075991 | China | 2016 | CN/Chongqing-148/2016 |
| KY075992 | China | 2016 | CN/Chongqing-150/2016 |
| KY075993 | China | 2016 | CN/Chongqing-155/2016 |
| KY075994 | China | 2016 | CN/Chongqing-156/2016 |
| KY418606 | China | 2016 | GD2016 |
| KY865242 | China | 2016 | CHN_Shanghai_0706_2016 |
| KY865243 | China | 2016 | CHN_Shanghai_0708_2016 |
| KY778776 | China | 2017 | CN/Shandong-1/201703 |
| KY778777 | China | 2015 | CN/Shandong-2/201703 |
| MG014377 | Germany | 2015 | DE2.8 |
| MG014362 | Germany | 2015 | DE3.7 |
| MG014363 | Germany | 2015 | DE4.3 |
| MG014378 | Germany | 2015 | DE5.15 |
| MG014379 | Germany | 2015 | DE6.1 |
| MG014364 | Germany | 2015 | DE7.3 |
| MG014380 | Germany | 2015 | DE12.19 |
| MG014365 | Germany | 2015 | DE13.20 |
| MG014381 | Germany | 2015 | DE14.15 |
| MG014382 | Germany | 2015 | DE15.19 |
| MG014383 | Germany | 2015 | DE17.20 |
| MG014366 | Germany | 2015 | DE18.2 |
| MG014367 | Germany | 2015 | DE19.15 |
| MG014368 | Germany | 2015 | DE23.17 |
| MG014369 | Germany | 2015 | DE26.17 |
| MG014370 | Germany | 2015 | DE27.16 |
| MG014384 | Germany | 2015 | DE28.12 |
| MG014385 | Germany | 2015 | DE31.17 |
| MG014371 | Germany | 2015 | DE34.5 |
| MG014372 | Germany | 2015 | DE41.16 |
| MG014373 | Germany | 2015 | DE48.7 |
| MG014374 | Germany | 2015 | DE52.18 |
| MG014375 | Germany | 2015 | DE53.8 |
| MG014376 | Germany | 2015 | DE55.1 |
| KY996337 | South Korea | 2016 | KU-1601 |
| KY996338 | South Korea | 2016 | KU-1602 |
| KY996339 | South Korea | 2016 | KU-1603 |
| KY996340 | South Korea | 2016 | KU-1604 |
| KY996341 | South Korea | 2016 | KU-1605 |
| KY996342 | South Korea | 2016 | KU-1606 |
| KY996343 | South Korea | 2016 | KU-1607 |
| KY996344 | South Korea | 2016 | KU-1608 |
| KY996345 | South Korea | 2016 | KU-1609 |
| KX458235 | USA | 2015 | 2164 |
| KX778720 | USA | 2015 | MO2015 |
| KT869077 | USA | 2015 | 29160 |
| KX898030 | USA | 2016 | MN2016 |
| KX966193 | USA | 2016 | SD2016 |
